# Suppression of Cortical Microtubule Reorientation and Stimulation of Cell Elongation in Arabidopsis Hypocotyls under Microgravity Conditions in Space

**DOI:** 10.3390/plants11030465

**Published:** 2022-02-08

**Authors:** Shiho Kato, Mana Murakami, Ryo Saika, Kouichi Soga, Kazuyuki Wakabayashi, Hirofumi Hashimoto, Sachiko Yano, Shohei Matsumoto, Haruo Kasahara, Motoshi Kamada, Toru Shimazu, Takashi Hashimoto, Takayuki Hoson

**Affiliations:** 1 Department of Biology, Graduate School of Science, Osaka City University, Osaka 558-8585, Japan; sha_ztmb_6p5rit_duk@yahoo.co.jp (S.K.); 5k.s2.nm@gmail.com (M.M.); lemondesk9@gmail.com (R.S.); soga@osaka-cu.ac.jp (K.S.); kazuwakaba@osaka-cu.ac.jp (K.W.); 2 Institute of Space and Astronautical Science, Japan Aerospace Exploration Agency, Sagamihara 252-5210, Japan; hashimoto.hirofumi@jaxa.jp; 3 Japan Aerospace Exploration Agency, Tsukuba 305-8505, Japan; yano.sachiko@jaxa.jp; 4 Japan Manned Space Systems, Tokyo 100-0004, Japan; shouhei582000@gmail.com (S.M.); kasahara.haruo@jaxa.jp (H.K.); 5 Advanced Engineering Services, Tsukuba 305-0032, Japan; kamada.motoshi@jaxa.jp; 6 Japan Space Forum, Tokyo 101-0062, Japan; shimazu@jsforum.or.jp; 7 Division of Biological Science, Nara Institute of Science and Technology, Ikoma 630-0192, Japan; hasimoto@bs.naist.jp

**Keywords:** Arabidopsis, cell elongation, cortical microtubules, gravity resistance, microgravity, Resist Tubule, space, tubulin

## Abstract

How microgravity in space influences plant cell growth is an important issue for plant cell biology as well as space biology. We investigated the role of cortical microtubules in the stimulation of elongation growth in Arabidopsis (*Arabidopsis thaliana*) hypocotyls under microgravity conditions with the Resist Tubule space experiment. The epidermal cells in the lower half of the hypocotyls of wild-type Columbia were longer in microgravity than at on-orbit 1 *g*, which precipitated an increase in the entire hypocotyl length. In the apical region, cortical microtubules adjacent to the outer tangential wall were predominantly transverse to the long axis of the cell, whereas longitudinal microtubules were predominant in the basal region. In the 9th to 12th epidermal cells (1 to 3 mm) from the tip, where the modification of microtubule orientation from transverse to longitudinal directions (reorientation) occurred, cells with transverse microtubules increased, whereas those with longitudinal microtubules decreased in microgravity, and the average angle with respect to the transverse cell axis decreased, indicating that the reorientation was suppressed in microgravity. The expression of tubulin genes was suppressed in microgravity. These results suggest that under microgravity conditions, the expression of genes related to microtubule formation was downregulated, which may cause the suppression of microtubule reorientation from transverse to longitudinal directions, thereby stimulating cell elongation in Arabidopsis hypocotyls.

## 1. Introduction

A proper structural form is essential for plant prosperity. The form of the whole plant reflects the sum of the rate and direction of growth of the constituting organs, and the direction of cell expansion is important for determining the shape of each organ. Cortical microtubules are assumed to be responsible for anisotropic cell expansion [1,2,3,4,5] by directing the orientation of the cellulose microfibrils [1,2,6,7] and by some additional mechanisms [1,2,8].

Plants are surrounded by a variety of environmental signals, such as light, gravity, temperature, and water, which strongly influence cell expansion. Gravity is always present in a constant direction and magnitude on Earth; therefore, plants have utilized gravity as the most stable and reliable signal for regulating cell expansion. Centrifugal acceleration has been shown to suppress cell elongation and promote lateral cell expansion [9], and under hypergravity conditions, the orientation of cortical microtubules is also affected. In the epidermis of azuki bean epicotyls grown at 1 *g*, cells with transverse cortical microtubules were predominant, but as gravitational acceleration increased, the percentage of cells with transverse microtubules decreased, whereas those with longitudinal microtubules increased [9]. On the other hand, the stem organs of tubulin mutants were shorter and thicker than those of the wild-type and showed helical growth at 1 *g*. A high degree of this twisting phenotype has been observed under hypergravity conditions [10]. Hypergravity also accelerates the reorganization of cortical microtubules into parallel arrays in *Brassica napus* protoplasts [11,12]. The expression of most α- and β-tubulin genes in Arabidopsis hypocotyls is upregulated under these conditions [13,14]. In addition, hypergravity induced some characteristic changes in the expression of microtubule-associated protein (MAP) genes, such as a transient increase in the expression of γ-tubulin and katanin genes [15,16], and a decrease in the levels of 65 kDa microtubule-associated protein-1 [17]. These results suggest that cortical microtubules are involved in the modification of anisotropic cell expansion in response to hypergravity stimuli.

The results of hypergravity experiments raise an important question as to whether cortical microtubules play a role in regulating anisotropic cell expansion in response to 1 *g* gravity, similar to that of hypergravity. To determine this, space experiments for obtaining microgravity control are needed, since it is impossible to produce true microgravity conditions for a sufficient duration on Earth. Under microgravity conditions in space, stimulation of elongation growth was observed in coleoptiles and roots of rice [18,19], and hypocotyls and inflorescence stems of Arabidopsis [20,21,22]. In Arabidopsis hypocotyls, microgravity stimulates cell elongation but suppresses lateral cell expansion [23]. Cortical microtubule dynamics in epidermal cells of Arabidopsis hypocotyls have been examined in space, using lines in which microtubules were visualized by labeling tubulin or MAPs with green fluorescent protein (GFP), and showed that the percentage of cells with transverse microtubules increased under microgravity conditions [23]. However, the analysis was limited to the subapical elongation region, and a comparison was made between ground control and microgravity, because of various constraints and limitations for microscopy in orbit.

In the present study, we analyzed the relationship between cell elongation and the orientation of cortical microtubules for each cell number from the tip to the base along the epidermal cell file of Arabidopsis hypocotyls, which were cultivated under either artificial 1 *g* or microgravity conditions on the Kibo Module of the International Space Station, chemically fixed in orbit, and returned to Earth (Resist Tubule space experiment). Combined with information on the modification of the expression of genes related to microtubule formation under microgravity conditions, we discuss the role of cortical microtubules in the regulation of anisotropic cell expansion in response to 1 *g* gravity.

## 2. Results

### 2.1. Hypocotyl Growth

The germination and seedling growth of wild-type Arabidopsis Columbia under artificial 1 *g* and microgravity conditions in the Cell Biology Experiment Facility (CBEF) on the Kibo were similar to those on Earth (Figure S1). Hypocotyls and roots elongated approximately along the gravity vector at on-orbit 1 *g*, whereas they tended to elongate in various directions under microgravity conditions, as reported previously [23,24].

**Figure 1:**
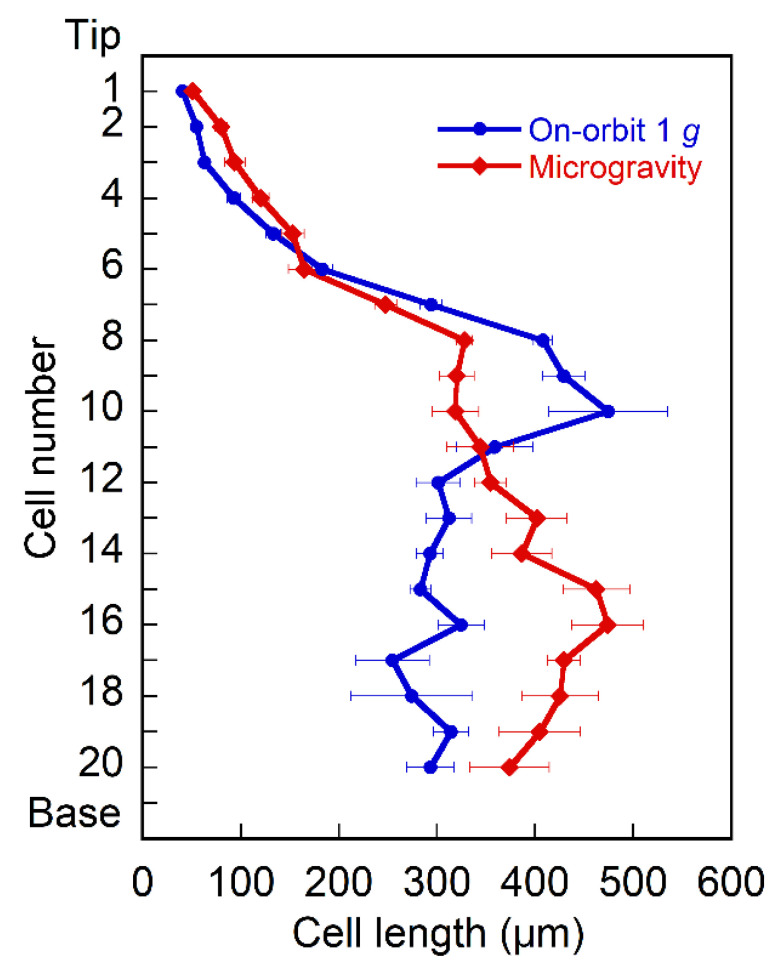
The length of epidermal cells in Arabidopsis hypocotyls. Wild-type Columbia seedlings were grown at 23.0 °C for 92 h under either on-orbit 1 *g* or microgravity conditions in the CBEF on the Kibo, fixed with an aldehyde mixture in orbit, and then returned to Earth, as described in Materials and Methods. The length of individual cells from the tip (cell 1) to the base (cell 20) was measured with a fluorescence microscope. Values are means ± SE (*n* = 20). Values are significantly different between the on-orbit control and microgravity (*p* < 0.05), except for cells 5, 6, 11, and 20.

The epidermal cell file of Arabidopsis hypocotyls consists of 20 cells [25]. The length of epidermal cells increased from the tip to the 8th cell, peaked from cells 8 to 10, decreased from cells 10 to 12, and thereafter remained primarily constant at on-orbit 1 *g* (Figure 1). Under microgravity conditions, the cell length gradually increased from the tip to the 13th cell, and then remained essentially constant at the base. At 1 *g*, cells 7 to 10 were comparatively longer, whereas cells 12 to 19 were relatively shorter (Figure 1), accounting for a 16% increase in whole hypocotyl length under microgravity conditions.

### 2.2. Orientation of Cortical Microtubules

**Figure 2:**
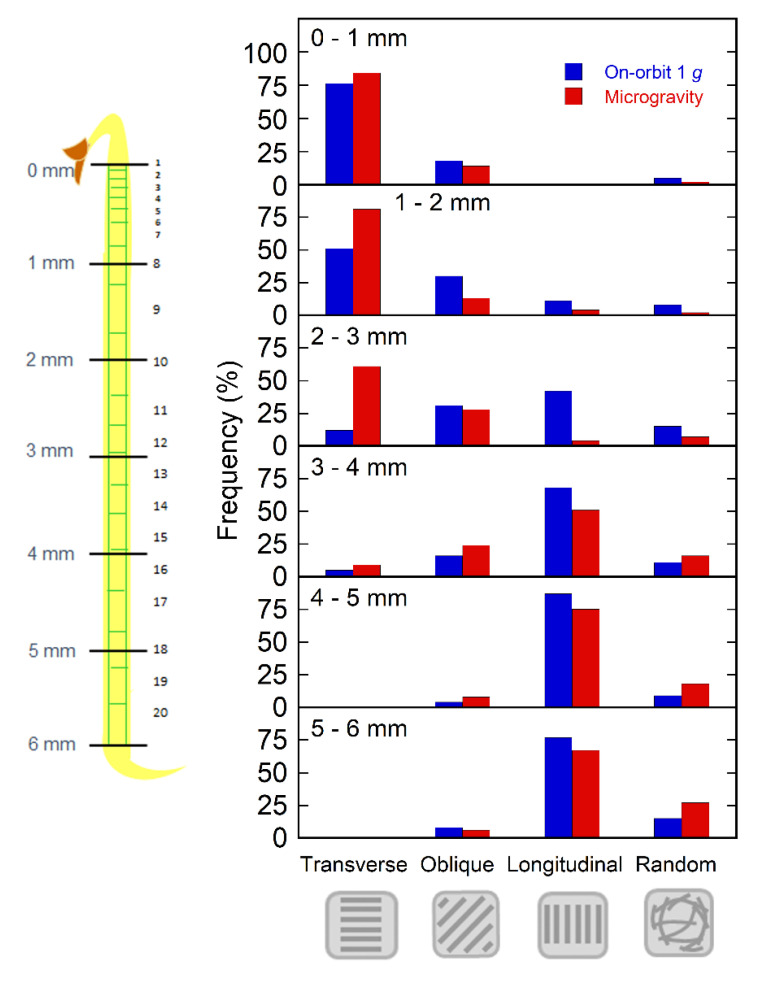
The orientation of cortical microtubules in epidermal cells of Columbia hypocotyls. Seedlings were grown as described in Figure 1 and cortical microtubules were stained and observed as described in Materials and Methods. The orientation of cortical microtubules adjacent to the outer tangential wall of the epidermal cell was determined. The frequency (percentage) of cells with cortical microtubules within a range of 0–30° (transverse), 30–60° (oblique), or 60–90° (longitudinal) with respect to the transverse cell axis, and in a variety of directions (random) was calculated for 200 cells in every 1 mm region from the tip to the base. In 1–2 and 2–3 mm regions, the proportion of four categories of cortical microtubule orientation was significantly different between the on-orbit control and microgravity (*p* < 0.05).

The orientation of cortical microtubules adjacent to the outer tangential wall of the epidermal cells was close to uniform in individual cells. Thus, we categorized cells into four types based on microtubule orientation: transverse, oblique, longitudinal, and random in every 1 mm region from the tip toward the base. In the apical region of hypocotyls, cortical microtubules were mostly transverse to the long axis of the cell, which favors elongation growth, whereas in the lower basal region, longitudinal microtubules, which induce lateral expansion, were predominant. There were no clear differences in microtubule orientation between the on-orbit 1 *g* control and microgravity samples in the apical 1 mm or lower 3–6 mm regions (Figure 2). However, at 1 to 3 mm from the tip, where the modification of microtubule orientation from transverse to longitudinal directions occurred, the percentage of cells with transverse microtubules increased under microgravity conditions, whereas that of longitudinal microtubules decreased (Figure 2 and Figure S2). In the 1–2 and 2–3 mm regions, the proportion of the four categories of cortical microtubule orientation was significantly different between the on-orbit control and the microgravity state (*p* < 0.05). These data support the results of a comparison between microgravity and ground control using GFP-expressing lines [23].

**Figure 3:**
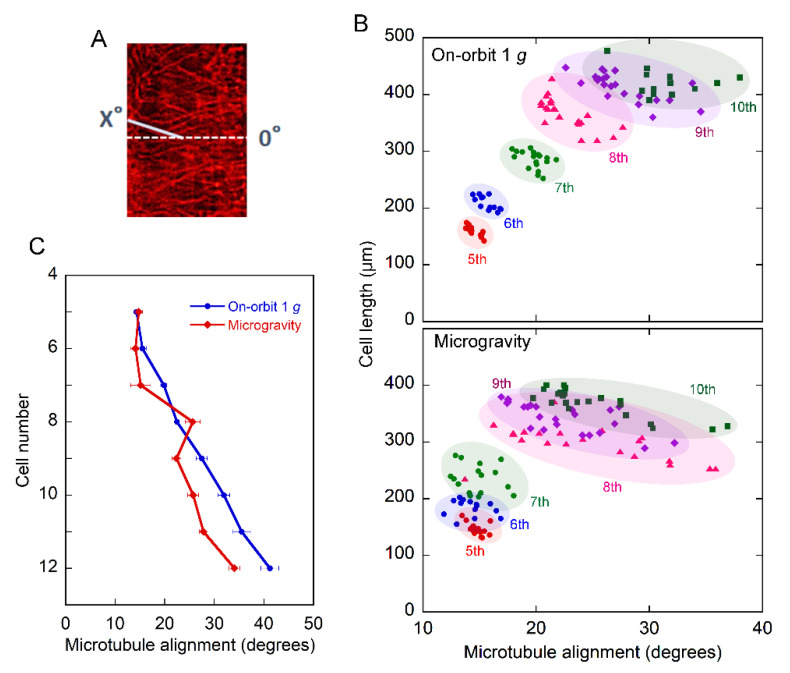
The alignment of cortical microtubules in each cell number of the epidermal cell file. Columbia seedlings were grown and their cortical microtubules were observed as described in Materials and Methods: (**A**) The transverse orientation to the long axis of the cell was defined as 0° and the angle of each cortical microtubule was measured for each cell; (**B**) The relationship between the cell length and the alignment of cortical microtubules in each cell of each cell number in the epidermal cell file. The cell length was measured as shown in Figure 1 and the alignment of cortical microtubules (average of 200 measurements) in individual cells as in Figure 3A; (**C**) Changes in the average alignment of cortical microtubules along the epidermal cell file. Data were taken from 14–24 different cells in each cell number and shown as means ± SE (*n* = 14–24). Values are significantly different between the on-orbit control and microgravity (*p* < 0.05), except for cells 5, 6, and 8.

We further confirmed this result by detailed analysis, in which the angle of each cortical microtubule to the transverse standard line was measured for every cell of each cell number (Figure 3A). Figure 3B shows the relationship between the cell length and microtubule alignment in the epidermal cell file. Both the cell length and angle of the microtubules gradually increased toward the base. Within the same cell number, there was a trend in that the smaller the microtubule angle, the longer the cell, both under 1 *g* and microgravity conditions. In addition, the angle of microtubules tended to be smaller and more variable under microgravity conditions than at 1 *g*.

The average angle of the microtubules gradually increased from cells 5 to 12 (Figure 3C). There was no clear trend in the differences in the angles between the on-orbit 1 *g* and microgravity samples up to the 8th cell. However, in cells 9 to 12, corresponding to the 1 to 3 mm regions, the angle was clearly smaller, clarifying that the microtubules were more transverse in microgravity than in the on-orbit control. These results indicate that the reorientation of microtubules from transverse to longitudinal directions is suppressed under microgravity conditions.

### 2.3. Expression of Tubulin and MAPs

**Figure 4:**
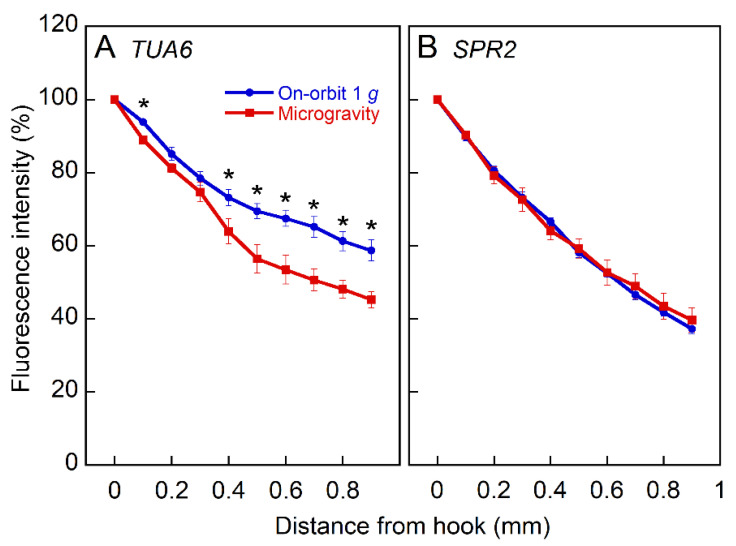
The expression of TUA6 and SPR2 in Columbia hypocotyls. TUA6pro-GFP and SPR2-GFP seedlings were grown at 23.0 °C for 72 h under either on-orbit 1 *g* or microgravity conditions in the CBEF on the Kibo. The fluorescence image was observed with the CB microscope, and fluorescence intensity of each 100 μm region was determined using ImageJ software. The expression level was normalized to the intensity value of the tip. *, Mean values with significant differences between the on-orbit control and microgravity (*n* = 10, *p* < 0.05).

The effect of microgravity on the expression of α-tubulin 6 (TUA6) and SPIRAL2 (SPR2), one of the MAPs, was examined with GFP-expressing Arabidopsis lines, a TUA6 promoter and GFP fusion reporter gene construct (TUA6pro-GFP) [26], and SPR2-GFP-expressing with a native SPR2 promoter (SPR2-GFP) [27]. The expression levels of both TUA6 and SPR2 were high in cotyledons and apical hook regions of hypocotyls, and decreased toward the base. As the absolute fluorescence intensity varied widely by sample, we quantified the relative fluorescence intensity along the hypocotyls (Figure 4). The relative intensity of TUA6 expression decreased toward the base, and this decrease was greater in microgravity than in the on-orbit control. However, microgravity had no effects on the expression of SPR2.

**Figure 5:**
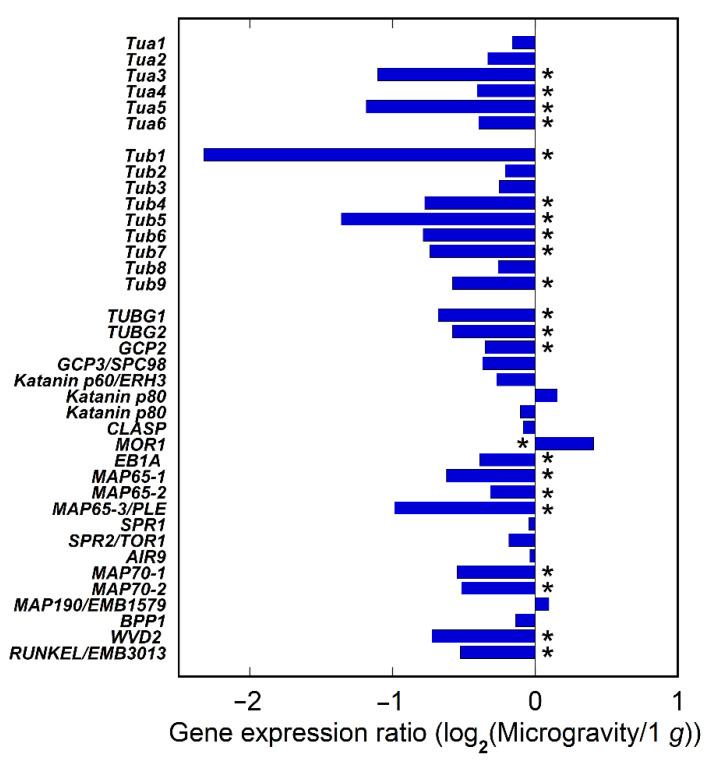
The effect of microgravity on expression of α-tubulin, ß-tubulin, and MAPs genes in the apical region of inflorescence stems in *tua4* mutant. The gene expression was shown as the ratio of expression under microgravity conditions to that of the on-orbit control. *, Mean values with significant differences between the on-orbit control and microgravity (*n* = 3, *p* < 0.05).

RNAseq analyses were conducted to confirm changes in the expression of genes related to microtubule formation under microgravity conditions. We used the apical regions of inflorescence stems from an amino acid substitution mutant of α-tubulin 4 (R2K, *tua4*) [28], obtained by a long-term experiment, as an alternative for this analysis, because the amount of material obtained from space-grown hypocotyls was limited and the mutant was hypersensitive to gravitational acceleration [10]. Figure 5 shows the ratio of the expression of major microtubule-related genes under microgravity conditions to that of the on-orbit control. The expression of four out of six α-tubulin genes and six out of nine β-tubulin genes was downregulated, whereas none was upregulated by microgravity. In contrast, the expression levels of MAPs were lower or higher, depending on their type, under microgravity conditions.

## 3. Discussion

The epidermal cells in the lower half of Columbia hypocotyls were longer in microgravity than at on-orbit 1 *g* (Figure 1), which precipitated a 16% increase in the entire hypocotyl length. Thus, stimulation of elongation growth under microgravity conditions in space was confirmed at the cellular level. On the other hand, in the apical region of Columbia hypocotyls, cortical microtubules adjacent to the outer tangential wall in epidermal cells were predominantly transverse to the long axis of the cell, whereas cells with longitudinal microtubules were predominant in the basal region, irrespective of gravity conditions (Figure 2). In the region of reorientation of cortical microtubules from transverse to longitudinal directions, the 9th to 12th cells from the tip, the percentage of cells with transverse microtubules increased, whereas that of their longitudinal counterparts decreased under microgravity conditions (Figure 2 and Figure S2). Thus, the average angle of microtubules with respect to the transverse cell axis decreased in this region (Figure 3C), indicating that the reorientation of cortical microtubules from transverse to longitudinal directions was suppressed under microgravity conditions.

The maintenance of transverse orientation of cortical microtubules under microgravity conditions (Figure 2 and Figure 3) may be related to stimulation of elongation growth (Figure 1), because microgravity stimulates cell elongation but suppresses lateral cell expansion in Arabidopsis hypocotyls [23]. However, in cells 9 to 12, cortical microtubules were more transverse, but cells were shorter in microgravity than in the on-orbit control. The lack of a clear-cut correlation between microtubule alignment and cell elongation has often been reported [29], and would be caused by the time lag between the set of microtubule orientation and induction of actual growth, and by the fact that growth is not uniform along the plant stem organs [25,30]. In rapidly elongating hypocotyls, a tipward wave of elongation occurs and expansion continues into the upper region [30]. As the Columbia hypocotyls used in this study were linearly elongating, the maintenance of transverse microtubule orientation would stimulate future cell elongation in cells 9 to 12 under microgravity conditions. The increase in cell length in the lower half of hypocotyls in microgravity may also be caused by such a mechanism. The fact that cortical microtubules were more transverse in longer cells within the same cell number (Figure 3B) supports this view. In the root transition zone, growth polarity is regulated by the interaction between microtubules and actin filaments [31], which is modified on the clinostat [32]. As there are various differences in growth properties between hypocotyls and roots, and in the effects of true microgravity and clinorotation on growth, future space experiments with roots are highly expected.

The analysis with GFP-expressing lines showed that the expression of TUA6 gene in hypocotyls was downregulated in microgravity (Figure 4). In the apical regions of inflorescence stems, the expression of most α-tubulin and β-tubulin genes was downregulated under microgravity conditions (Figure 5), as was the expression of some MAP genes, such as components of the γ-tubulin complex. These data confirmed the results of preliminary microarray analysis between ground control and microgravity [22]. It has been proposed that the cytosolic γ-tubulin complex binds to the side of preexisting cortical microtubules and nucleates new microtubules as branches by recruiting α- and β-tubulins, resulting in modification of orientation of the cortical microtubule array [33,34]. The present results are consistent with this mechanism and with the suppression of cortical microtubule reorientation in microgravity. In addition, a recent phosphoproteome showed that the phosphorylation of α-tubulins, which stimulates microtubule depolymerization, was suppressed under microgravity conditions [35]. These results suggest that under microgravity conditions, the expression of genes related to microtubule formation was downregulated, which may cause the suppression of microtubule reorientation from transverse to longitudinal directions, thereby stimulating cell elongation in Arabidopsis hypocotyls. In the present analysis, we used the apical parts of inflorescence stems, as opposed to hypocotyls, because the amount of materials obtained from space-grown hypocotyls was limited. However, it is obvious that there are numerous differences in cell types, age, growth properties, and growth conditions, between hypocotyls and inflorescence stems. We, therefore, should be careful to evaluate the present RNAseq analysis data. Further transcriptome analysis with seedlings grown in microgravity, as conducted by other research groups [35,36], is needed to clarify these results.

Mechanical resistance to gravitational acceleration is a principal graviresponse in plants, comparable to gravitropism. This response has been termed ‘gravity resistance’ and its nature and mechanism have been examined by ground-based experiments using centrifugal hypergravity conditions [37,38]. The results have shown that modification of growth anisotropy, namely the suppression of elongation growth and stimulation of lateral expansion, by the reorientation of cortical microtubules from transverse to longitudinal directions, contributes to plant resistance to hypergravity [9,10]. However, the role of cortical microtubules in resistance to 1 *g* gravity remained unclear. In the present study, we showed that under microgravity conditions, the reorientation of cortical microtubules from transverse to longitudinal directions was suppressed, thereby stimulating cell elongation in Arabidopsis hypocotyls. These results, in combination with the fact that longitudinal growth of the inflorescence stems of dominant-negative α-tubulin mutants was restored to the wild-type level under microgravity conditions [22], supports the hypothesis that cortical microtubules play a role in the gravity resistance of plants to 1 *g* gravity. Mid1-complementing activity (MCA) proteins, candidates for mechanosensitive ion channels, are involved in the perception of hypergravity signals and may be responsible for plant resistance to hypergravity [39]. The role of MCAs in the perception of gravity signals at 1 *g* is yet to be determined.

Live imaging in orbit is ideal for clarifying the dynamics of cortical microtubules under microgravity conditions. However, there are various difficulties and limitations for microscopy in orbit; for instance, the preparation of glass slide sets in microgravity is challenging, and the operation of the microscope by directing from the ground can be problematic [23,40]. In particular, since it takes more than 30 min from discontinuing the rotation of the CBEF turntable for the removal of plant materials, to the start of microscopic observation, on-orbit 1 *g* control is not possible. Thus, in the recent Aniso Tubule space experiment using GFP-expressing Arabidopsis lines, we had to narrow down the subapical elongation region and compare microgravity samples with the ground control [23]. On the other hand, in the present study using Arabidopsis hypocotyls, which were cultivated under either artificial 1 *g* or microgravity conditions and chemically fixed in orbit, we analyzed in detail the orientation of cortical microtubules for every cell of each cell number from the tip to the base along the epidermal cell file. However, this procedure is not always suitable for understanding the dynamics, and possible disruptions due to chemical fixation or transport from space to ground cannot be excluded. A novel centrifugal microscope with a confocal instrument should be developed for space experiments to confirm the present results.

## 4. Materials and Methods

### 4.1. Plant Materials and Onboard Experiments

Surface-sterilized seeds of wild-type *Arabidopsis thaliana* (L.) Heynh. ecotype Columbia (Col-0) were sown in the cellulose nonwoven fabric of a newly designed Cultivation Chamber [40], which was then launched on the SpaceX CRS-1 spacecraft. In orbit, the Chambers were unstowed, and initial watering of seeds was carried out manually with a syringe. The Chambers containing seeds were kept at 2 °C for 4 d, and then set in the Video Measurement Unit (V-MEU) installed in the CBEF in the Kibo Module of the International Space Station. One V-MEU was placed in the microgravity compartment, and the other was rotated to produce acceleration at 1 *g*. Seedlings were grown at 23 °C in the dark, after white light illumination by LEDs for 6 h to induce germination. After 92 h, seedlings were removed from the Chamber and fixed with a mixture of 1.5% (*v*/*v*) formaldehyde and 0.5% (*v*/*v*) glutaraldehyde in PEMT buffer (50 mM PIPES, 2 mM EGTA, 2 mM MgSO_4_, 0.05% (*v*/*v*) Triton X-100, pH 7.2) in the Kennedy Space Center Fixation Tubes (KFTs). The KFTs were then kept at 2 °C in the Minus Eighty-Degree Laboratory Freezer for ISS (MELFI), returned to Earth on SpaceX-1 Dragon spacecraft, and transported to the laboratory of Osaka City University for microscopic analyses.

In another experiment, surface-sterilized seeds of GFP-expressing lines of Arabidopsis, TUA6pro-GFP and SPR2-GFP, were sown in the cellulose nonwoven fabric of the Cultivation/Observation Chamber [23,40], which was then launched on the SpaceX CRS-3 and Cygnus CRS Orbital-2 spacecrafts. In orbit, the Chambers were unstowed, and initial watering of seeds was carried out manually using a syringe. The Chambers containing seeds were kept at 2 °C for 4 d, and then set in the V-MEU installed in the CBEF in the Kibo Module. One V-MEU was placed in the microgravity compartment, and the other was rotated to produce acceleration at 1 *g*. Seedlings were grown at 23 °C in the dark, after white light illumination by LEDs for 6 h to induce germination. After 72 h, the chamber was removed from the CBEF, and distilled water was injected with a syringe into the chamber for microscopic observation. The interior depth of the Chamber was reduced by tightening the screw in the Chamber. Then, the Chamber was set on the CB microscope (https://iss.jaxa.jp/en/kiboexp/pm/cb/, last accessed on 16 January 2022) equipped with filter sets to detect GFP. Microscopic operations were controlled from the ground by command. Micrographs were saved and downlinked to the earth in real time or with a minimum delay. The intensity of GFP was determined using ImageJ software (http://rsbweb.nih.gov/ij/, last accessed on 16 January 2022).

In a long-term experiment, surface-sterilized seeds of *tua4* mutant [28] were adhered to a seedbed of rock wool with 1% (*w*/*v*) gum arabic in the Plant Chamber of the Plant Experiment Unit (PEU). The PEUs containing the seeds were launched on the HTV-4 cargo spacecraft, and in orbit, they were unstowed and installed in the CBEF of the Kibo module. One PEU was placed in the microgravity compartment, and the other was rotated to produce acceleration at 1 *g*. After initial watering of seeds, germinated plants were grown at 23.5 ± 0.2 °C under continuous blue/red LED light at 110 μmol m^−2^ s^−1^. The relative humidity was maintained between 70% and 80%. After 39 d, Arabidopsis plants were removed from the Plant Chamber and fixed with RNAlater solution in a newly designed Chemical Fixation Bag (CFB) [40]. The CFBs containing fixed plant materials were frozen in the MELFI and recovered to Earth on the SpaceX CRS-3 spacecraft, and then transported to the laboratory of Osaka City University for gene expression analyses.

### 4.2. Microscopy

Whole seedlings, fixed in orbit and returned to Earth, were rinsed three times with PEMT buffer. They were subsequently treated with 0.05% (*w*/*v*) Pectolyase Y-23 (Kyowa Chemical Products, Osaka, Japan) and 0.05% (*w*/*v*) Cellulase Y-C (Kyowa Chemical Products) in PEMT buffer with 0.4 M mannitol for 20 min at 30 °C, and rinsed three times with PEMT buffer. After incubation in 0.5% Triton X-100 for 1 h, the seedlings were incubated with primary antibodies against α-tubulin (T6199; Sigma, St. Louis, MI, USA) diluted 1:1000 (*v*/*v*) in 1% BSA at 30 °C for 16 h, and rinsed three times with PEMT buffer. Seedlings were then incubated with Cy3-conjugated anti-mouse IgG (product C2181; Sigma) diluted 1:100 in (*v*/*v*) in 1% BSA at 37 °C for 3 h, and rinsed three times with PBS. They were mounted on a glass slide and covered with a solution containing 90% (*v*/*v*) glycerol. Immunofluorescence images were collected using a fluorescence microscope (Axio Imager A1; Carl Zeiss, Göttingen, Germany) equipped with a cooled CCD camera (VB-7000; Keyence, Osaka, Japan), and processed with bundled image processing software (BZ-H1A, Keyence).

The orientation of cortical microtubules adjacent to the outer tangential wall of the epidermal cells was determined from the processed images. Since the orientation was almost uniform in each cell, we measured the orientation of each cell from the tip to the base along the epidermal cell file. We determined the frequency of cells with cortical microtubules in the range of 0–30° (transverse), 30–60° (oblique), or 60–90° (longitudinal) with respect to the transverse cell axis, and in a variety of directions (random) in each 1 mm region from the tip. Cells with basket-patterned arrays [41] were counted as random. In the detailed analysis, the transverse orientation to the long axis of the cell was defined as 0° and the angle of each cortical microtubule was measured. The average angle was then calculated for each cell of each cell number.

### 4.3. Gene Expression Analysis

Inflorescence stem segments of *tua4* mutant, obtained by the long-term experiment, were immediately frozen in liquid nitrogen and stored at –80 °C until analysis. The apical 5 mm regions were cut and homogenized with a mortar and pestle. Total RNA was prepared using the RNeasy Plant Mini Kit (Qiagen, Valencia, CA, USA), including a DNA elimination step (RNase-Free DNase Set, Qiagen). Preparation of cDNA library and RNAseq analysis were performed using the custom service of Hokkaido System Science (Sapporo, Japan) with an Illumina paired-end HiSeq analysis system run on a HiSeq2000 sequencer (Illumina, San Diego, CA, USA). Contaminating Illumina adapter sequences and low-quality bases were trimmed using the manufacturer’s protocol. Paired-end 100-bp reads were aligned and annotated with Arabidopsis TAIR10 genome sequence data (https://www.arabidopsis.org, last accessed on 16 January 2022). Differential expression analysis was conducted using the Cuffdiff program (http://cole-trapnell-lab.github.io/cufflinks/, last accessed on 16 January 2022).

### 4.4. Statistical Analysis

For each measurement, the mean and standard error of the mean (SE) were calculated. The significance of differences between the on-orbit 1 *g* and microgravity was analyzed using the Student’s *t*-test. The difference in the proportion of the four categories of cortical microtubule orientation was analyzed using the Pearson’s chi-square test.

## 5. Conclusions

The present study, using true microgravity conditions in space as the control, supports the hypothesis that cortical microtubules play an essential role in the plant response to gravity in the range from 1 *g* to hypergravity.

## Data Availability

The data and materials that support the findings of this study are available from the corresponding author upon reasonable request.

## Supplementary Materials

The following supporting information can be downloaded at: https://www.mdpi.com/article/10.3390/plants11030465/s1, Figure S1: Columbia seedlings grown under on-orbit 1 *g* or microgravity conditions in the CBEF on the Kibo, Figure S2: Fluorescence images of arrays of cortical microtubules adjacent to the outer tangential wall of epidermal cells at 2 to 3 mm from the tip in Columbia hypocotyls grown under on-orbit 1 *g* or microgravity conditions.
