# Supplementary figures and images for: Suppression of Cortical Microtubule Reorientation and Stimulation of Cell Elongation in Arabidopsis Hypocotyls under Microgravity Conditions in Space
Source: Plants (Basel). 2022 Feb 8;11(3):465. doi: 10.3390/plants11030465 (PMC8837939; doi:10.3390/plants11030465)

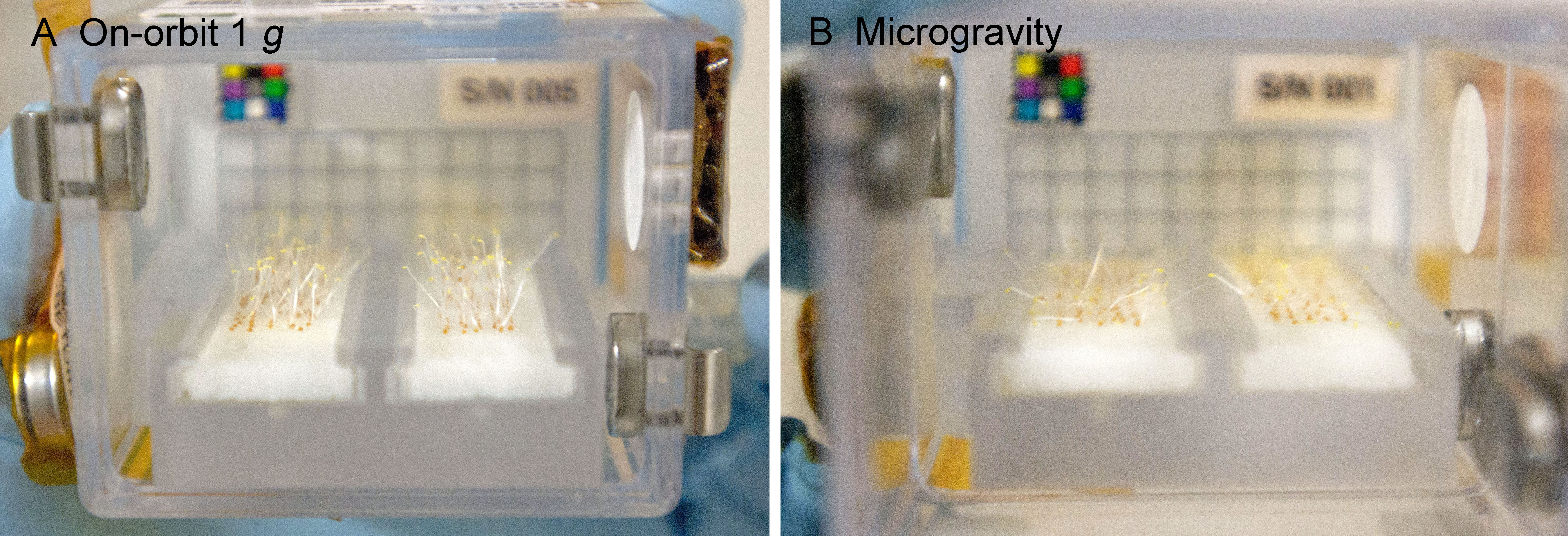

Supplement: Supplementary file 1 [file plants-11-00465-s001.zip › plants-1579390-suppl/Kato et al_FigS1.jpg]

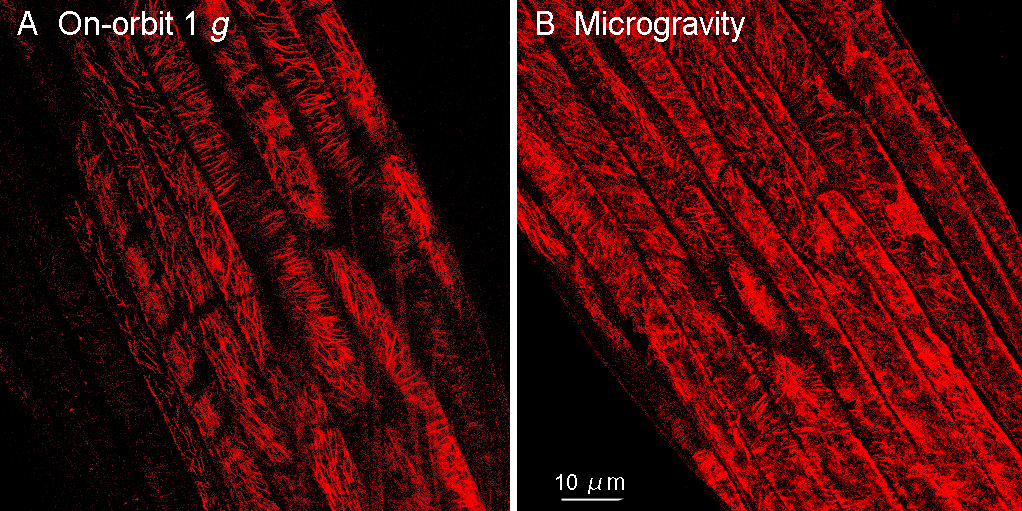

Supplement: Supplementary file 1 [file plants-11-00465-s001.zip › plants-1579390-suppl/Kato et al_FigS2.jpg]
